# Conditions of In Vitro Biofilm Formation by Serogroups of *Listeria monocytogenes* Isolated from Hass Avocados Sold at Markets in Mexico

**DOI:** 10.3390/foods10092097

**Published:** 2021-09-05

**Authors:** María Guadalupe Avila-Novoa, Velia Navarrete-Sahagún, Jean Pierre González-Gómez, Carolina Novoa-Valdovinos, Pedro Javier Guerrero-Medina, Ramón García-Frutos, Liliana Martínez-Chávez, Nanci Edid Martínez-Gonzáles, Melesio Gutiérrez-Lomelí

**Affiliations:** 1 Centro de Investigación en Biotecnología Microbiana y Alimentaria, Departamento de Ciencias Básicas, División de Desarrollo Biotecnológico, Centro Universitario de la Ciénega, Universidad de Guadalajara, Av. Universidad 1115, Ocotlán 47810, Jalisco, Mexico; avila.novoa@cuci.udg.mx (M.G.A.-N.); velia.nsahagun@alumnos.udg.mx (V.N.-S.); carolina.novoa@alumnos.udg.mx (C.N.-V.); pjgm@cuci.udg.mx (P.J.G.-M.); 2 Laboratorio Nacional para la Investigación en Inocuidad Alimentaria (LANIIA), Centro de Investigación en Alimentación y Desarrollo A.C. (CIAD), Carretera a Eldorado Km. 5.5, Culiacán 80110, Sinaloa, Mexico; jgonzalez.219@estudiantes.ciad.mx; 3 Departamentos de Farmacobiología y Matemáticas, CUCEI, Universidad de Guadalajara, Marcelino García Barragán 1451, Guadalajara 44430, Jalisco, Mexico; ramon.garcia9266@alumnos.udg.mx (R.G.-F.); liliana.mchavez@academicos.udg.mx (L.M.-C.); nanci.martinez@academicos.udg.mx (N.E.M.-G.)

**Keywords:** *Listeria monocytogenes*, virulence, serogroups, biofilms, extracellular matrix, food surface contact

## Abstract

*Listeria monocytogenes* is an important pathogen that has been implicated in foodborne illnesses and the recall of products such as fruit and vegetables. This study determines the prevalence of virulence-associated genes and serogroups and evaluates the effects of different growth media and environmental conditions on biofilm formation by *L. monocytogenes*. Eighteen *L. monocytogenes* isolates from Hass avocados sold at markets in Guadalajara, Mexico, were characterized by virulence-associated genes and serogroup detection with PCR. All isolates harbored 88.8% *actA*, 88.8% *plcA*, 83.3% *mpl*, 77.7% *inlB*, 77.7% *hly*, 66.6% *prfA*, 55.5% *plcB*, and 33.3% *inlA*. The results showed that 38.8% of isolates harbored virulence genes belonging to *Listeria* pathogenicity island 1 (LIPI-1). PCR revealed that the most prevalent serogroup was serogroup III (1/2b, 3b, and 7 (*n* = 18, 66.65%)), followed by serogroup IV (4b, 4d–4e (*n* = 5, 27.7%)) and serogroup I (1/2a–3a (*n* = 1, 5.5%)). The assessment of the ability to develop biofilms using a crystal violet staining method revealed that *L. monocytogenes* responded to supplement medium TSBA, 1/10 diluted TSBA, and TSB in comparison with 1/10 diluted TSB (*p* < 0.05) on polystyrene at 240 h (*p* < 0.05). In particular, the biofilm formation by *L. monocytogenes* (7.78 ± 0.03–8.82 ± 0.03 log_10_ CFU/cm^2^) was significantly different in terms of TSBA on polypropylene type B (PP) (*p* < 0.05). In addition, visualization by epifluorescence microscopy, scanning electron microscopy (SEM), and treatment (DNase I and proteinase K) revealed the metabolically active cells and extracellular polymeric substances of biofilms on PP. *L. monocytogenes* has the ability to develop biofilms that harbor virulence-associated genes, which represent a serious threat to human health and food safety.

## 1. Introduction

*Listeria monocytogenes* is a bacterium ubiquitous in the environment and is the causative agent of listeriosis, leading to septicemia, encephalitis, endocarditis, meningitis, abortions, and stillbirths [1,2]. The severity of the pathology is associated with several at-risk groups, such as those with a weak immune system, adults >65 years, pregnant women, and newborn babies [3,4]. *Listeria*, *Salmonella*, and Shiga toxin-producing *Escherichia coli* (STEC) are the most common causes of hospitalizations (82%) and deaths (825) reported in outbreaks with a single confirmed etiology [5].

According to the Interagency Food Safety Analytics Collaboration (IFSAC) in 2018, foodborne *L. monocytogenes* illnesses are mostly attributed to fruit (32.5%) and vegetable row crops (11.6%) [6]. In particular, the FDA (2020–2021) issues recalls of vegetable products and fruits when they are associated with a potential risk of *L. monocytogenes* [7]. A recent sampling (May 2014 to November 2015) by the FDA demonstrated the prevalence of *L. monocytogenes* in samples of avocado fruit pulp (0.24%) and skin (17.33%) [8]. Clearly, avocados have a high lipid (saturated, polyunsaturated, and monounsaturated fatty acids) and moisture content, a non-acidic pH level, and low carbohydrates, which can make them a favorable growth medium for *L. monocytogenes*. In addition, the fresh produce supply chain is complex, with increasing potential sources of *L. monocytogenes* contamination. For example, contamination from irrigation water, fertilization with contaminated manure and contaminated soil (agricultural practices), cross-contamination from surfaces contaminated with pathogens, food spoilage, improper manipulation by food handlers, or processing fresh produce into fresh-cut products increases the risk of bacterial growth by breaking the fruit’s skin and allowing for the spread and potential growth of any pathogens that may be present [3,8,9,10,11,12].

Determining the virulence potential of *L. monocytogenes* is important in terms of public health, as is risk identification in ready-to-eat (RTE) fruits and vegetables because they provide critical information regarding food sources of listeriosis [13]. Currently, *L. monocytogenes* consists of four major evolutionary lineages (I, II, III, and rare lineage IV), including 14 recognized serotypes of *L. monocytogenes* that are grouped into four PCR serogroups [13,14,15,16]. In addition, *L. monocytogenes* has the ability to develop a biofilm and is thus persistent in food-processing environments and can be a subsequent source of contamination, representing a serious concern for human health and food safety [1,11,17,18,19,20,21]. Biofilm confers advantages to pathogens and food spoilage, such as desiccation resistance, as well as UV and chemical protection [18,22,23].

Previous research has demonstrated the persistence of *L. monocytogenes* associated with biofilm formation; however, it is a complex system regulated by environmental and genetic factors of the strains. In fact, extracellular polymeric substances (EPS), apart from contributing to adhesion to the surface and for cohesion in the biofilm, have the function of protecting individual cells from environmental stress [20].

Therefore, it is important to know the behavior of *L. monocytogenes* in the environment and in components of the biofilm matrix and to generate new strategies for the prevention or removal of biofilm from fresh produce. It is also critical to determine the prevalence of serogroups of *L. monocytogenes* and their virulence potential, which is necessary to understand their diversity. Hence, the objectives of this research were (i) to determine the prevalence of virulence-associated genes and serogroups of *L. monocytogenes* from Hass avocados, (ii) to evaluate the effects of different growth media and environmental conditions on biofilm formation by *L. monocytogenes*, and (iii) to determine the components of the extracellular matrix in *L. monocytogenes* biofilm.

## 2. Materials and Methods

### 2.1. L. monocytogenes Isolates

In total, 18 *L. monocytogenes* isolates were evaluated in this study, which were previously obtained from Hass avocados sold at retail markets in Guadalajara, Mexico [12]. Stocks of *L. monocytogenes* were stored using the protocol described by Avila-Novoa et al. [24]. Working cultures of *L. monocytogenes* were maintained in tryptic soy broth (TSB; Becton Dickinson Bioxon, Le Pont de Claix, France) with 0.6% yeast extract (TSBYE) (Sigma-Aldrich, St. Louis, MO, USA) and confirmed by PCR.

### 2.2. PCR-Serogroup Analysis and Virulence Genes

Bacterial DNA was extracted from 24 h/30 °C cultures in TBSYE using the protocol described by Avila-Novoa et al. [24]. All *L. monocytogenes* strains were investigated for the detection of *prfA*, *plcA*, *hly*, *mpl*, *actA*, *plcB*, *inlA*, and *inlB* genes by PCR using the protocol by Montero et al. [25]. The amplification conditions used were as follows: 5 min at 94 °C; 35 cycles of 40 s at 94 °C, 75 s at different temperatures for different genes, and 75 s at 72 °C; followed by a final extension of 10 min at 72 °C (Table 1). Alternatively, the serogroups of *L. monocytogenes* isolates were determined by Doumith et al. [14] for the detection of the four PCR serogroups of *L. monocytogenes*, as described: I (1/2a and 3a), II (1/2c and 3c), III (1/2b, 3b, and 7), and IV (4b, 4d, and 4e). *L. monocytogenes* ATCC 19111 was used as the positive control.

### 2.3. Biofilm Formation Assay

The ability of the strains to form biofilms was assessed in four culture media (TSB, 1/10 diluted TSB, TSBA (TSB + 1% Hass avocado residues), 1/10 diluted TSBA (1/10 TSB + 1% Hass Avocado residues)) in polystyrene microtiter plates (Corning^®^ 96-Well Assay Microplate, Lowell, MA, USA) using crystal violet (CV) staining as described by Stepanović et al. [26] and Lee et al. [27], with some modifications. For each of the strains, 230 μL of each of the culture media (TSB, 1/10 diluted TSB, TSBA, 1/10 diluted TSBA) + 20 μL bacterial suspension (~10^8^ CFU/mL) were incorporated; this was done in triplicate. Wells filled with broth medium (TSB, 1/10 diluted TSB, TSBA, 1/10 diluted TSBA) were used as negative controls, and *L. monocytogenes* ATCC 19111 was used as the positive control. Then, the plates were incubated at 30 °C for 48 h and 240 h. Afterwards, the planktonic bacteria were removed with phosphate-buffered saline (PBS; 7 mM Na_2_HPO_4_, 3 mM NaH_2_PO_4_ and 130 mM NaCl, pH 7.4) and the biofilm was fixed with methanol for 5 min and stained with 100 µL of 0.1% crystal violet solution for 15 min. The excess stain was rinsed off with sterile water and resolubilized with 33% (*v/v*) glacial acetic acid. Absorbance was measured at 570 nm (OD_570_), using a Multiskan FC (Thermo Fisher Scientific, Inc., Madison, WI, USA). The cutoff OD (ODc) was defined using the protocol described by Stepanović et al. [26].

### 2.4. Conditions and Quantification of Mono-Species Biofilms

Biofilms were developed on PP coupons (polypropylene type B (2 × 0.7 × 0.2 cm; Plásticos Tarkus, Jalisco, Mexico)), using the protocol described by Avila-Novoa et al. [24] for 240 h at 30 °C in 10 mL of 4 different media: TSB, 1/10 diluted TSB, TSBA, and 1/10 diluted TSBA, and inoculated with 100 μL of the corresponding strain of *L. monocytogenes* (~10^8^ CFU/mL). The quantification was carried out as described by Avila-Novoa et al. [24]. *L. monocytogenes* ATCC 19111 was used as the positive control.

### 2.5. Epifluorescence Microscopy and Scanning Electron Microscopy (SEM)

After incubation at 30 °C for 240 h, the PP coupons were removed and processed using the protocol described by Avila-Novoa et al. [24]. Alternatively, SEM was performed using the methodology described by Borucki et al. [22] and Fratesi et al. [28]. Biofilms were observed using a TESCAN Mira3 LMU scanning electron microscope (Brno-Kohoutovice; Czech Republic). *L. monocytogenes* ATCC 19111 was used as the positive control and a PP coupon without inoculum was included in all assays.

### 2.6. Determination of Biofilm Components

#### 2.6.1. Matrix Characterization

Biofilm detachment assays were carried out as described by Fredheim et al. [29] and Avila-Novoa et al. [30]. Previously mature biofilms cultivated in TSB at 30 °C for 240 h in polystyrene microtiter plates were washed with 0.9% NaCl. The major components of *L. monocytogenes* biofilms were treated with (i) proteinase K (Promega, Madison, WI, USA) and (ii) 0.5 mg/mL DNase I (Roche, Mannheim, Germany) using the protocol described by Avila-Novoa et al. [24].

#### 2.6.2. Phenotype Analysis of Biofilm Production

All *L. monocytogenes* strains were characterized phenotypically by culture on Congo red agar (CRA) plates according to the protocol described by Arciola et al. [31], with some modifications. Briefly, CRA was prepared with TSBYE, 30 g/L of glucose (Sigma-Aldrich, St. Louis, MO, USA), 15 g/L of bacteriological agar (Becton Dickinson Bioxon, Le Pont de Claix, France), and 40 mg/L of Congo red (Sigma-Aldrich, Steinheim, Germany). According to the macroscopic characteristics developed by *L. monocytogenes* in the CRA, they were interpreted as (a) biofilm producers: black colonies with a dry filamentous, or (b) nonproducers: smooth pink colonies.

### 2.7. Statistical Analysis

All the experiments were performed in triplicate, and the data were evaluated using analysis of variance (ANOVA), followed by a Fisher’s least significant difference (LDS) test, using the Statgraphics Centurion XVI software program (StatPoint Technologies, Inc., Warrenton, VA, USA).

## 3. Results

### 3.1. Prevalence of Virulence Genes and Distribution of L. monocytogenes Serogroups Based on PCR Analysis

All isolates harbored 88.8% *actA*, 88.8% *plcA*, 83.3% *mpl*, 77.7% *inlB*, 77.7% *hly*, 66.6% *prfA*, 55.5% *plcB*, and 33.3% *inlA*. Additionally, 38.8% of *L. monocytogenes* isolates were positive for the *prfA*, *plcA*, *hly*, *mpl*, *actA*, and *plcB* genes associated with *Listeria* pathogenicity island 1 (LIPI-1) (Table 2). Based on variable gene content, 18 isolates were divided into serogroup I (1/2a–3a (*n* = 1, 5.5%)), serogroup III (1/2b, 3b, and 7 (*n* = 18, 66.65%)), and serogroup IV (4b, 4d–4e (*n* = 5, 27.7%)).

### 3.2. Ability to Form Biofilms

All isolates of *L. monocytogenes* had a higher biomass biofilm in TSBA (OD_570_ = 0.071–0.350, respectively) and 1/10 diluted TSBA (OD_570_ = 0.070–0.291) in comparison with 1/10 diluted TSB (OD_570_ = 0.066–0.135) (*p* < 0.05) at 48 h. Furthermore, there was no significant difference between TSBA and 1/10 diluted TSBA (*p* > 0.05), TSB and 1/10 diluted TSB (*p* > 0.05), and TSBA and TSB (*p* > 0.05) at 48 h. Clearly, the development of the biofilm of *L. monocytogenes* was favored at 240 h (*p* < 0.05). In general, *L. monocytogenes* had a higher biomass biofilm in TSBA (OD_570_ = 0.161–0.447) in comparison with TSB (OD_570_ = 0.155–0.302; *p* < 0.05) and 1/10 diluted TSB (OD_570_ = 0.141–0.262; *p* < 0.05) at 240 h; there was no difference between TSBA and 1/10 diluted TSBA (OD_570_ = 0.173–0.441; *p* > 0.05). In addition, *L. monocytogenes* had a higher biomass biofilm in 1/10 diluted TSBA in comparison with 1/10 diluted TSB *p* < 0.05); there was no significant difference between 1/10 diluted TSBA and TSB (*p* > 0.05) at 240 h.

Finally, *L. monocytogenes* strains were classified as strong biofilm producers (22%), moderate biofilm producers (5.5–22%), or weak biofilm producers (38.8–77.7%) depending on the composition of the medium culture at 48 h. The mean OD_570_ values obtained by the quantitative biofilm production of *L. monocytogenes* strains in the different medium culture are shown in Table 3. However, *L. monocytogenes* strains were classified only as strong (5.5–61.1%) or moderate biofilm producers (38.8–94.4%) at 240 h (Table 3).

Alternatively, four strains of *L. monocytogenes* (Lm-303, Lm-320, Lm-352, and Lm-356) were selected depending on the serogroup type, strong biofilm producers, and genetic profile (Table 2 and Table 3). Table 4 describes the development of mono-species models considering strains and culture media. All the tested microorganisms showed a strong ability to develop biofilms on PP serogroup III (serotype 1/2b, 3b, and 7) (Lm-352 (7.78 ± 0.03 log_10_ CFU/cm^2^) and Lm-356 (7.84 ± 0.30 log_10_ CFU/cm^2^)) and serogroup IV (serotype 4b, 4d, and 4e) (Lm-303 (8.36 ± 0.01 log_10_ CFU/cm^2^) and Lm-320 (8.82 ± 0.03 log_10_ CFU/cm^2^)) in TSBA (*p* < 0.05). In addition, biofilms developed by Lm-303 were present in lower amounts than Lm-320 (*p* < 0.05).

**Figure 1:**
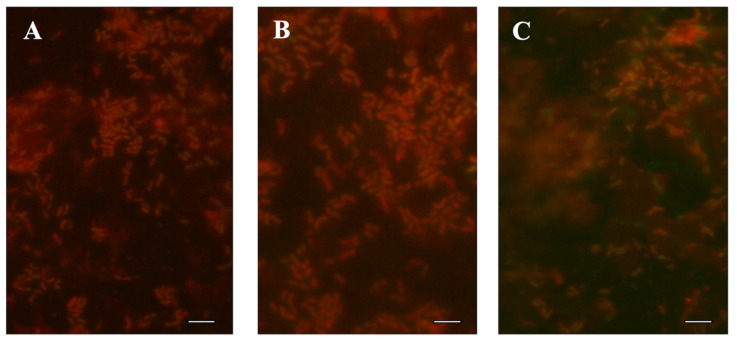
Micrographs of biofilm of *L. monocytogenes* development on polypropylene type B (PP) by epifluorescence microscopy. The biofilms were developed at 30 °C for 240 h. (**A**) Lm-303 TSB; (**B**) Lm-320 in 1/10 diluted TSB; (**C**) Lm-356 in TSBA. The white bar scale indicates 5 µm.

**Figure 2:**
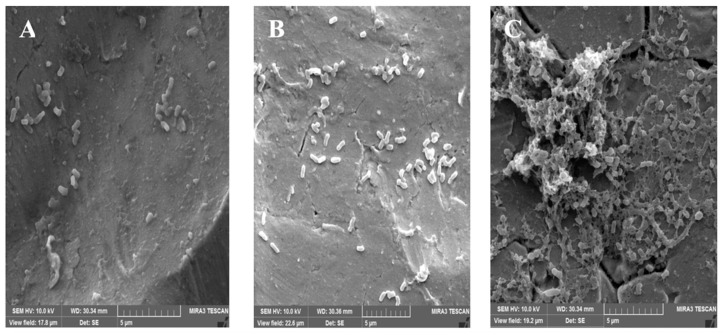
Micrographs of biofilm of *L. monocytogenes* development on polypropylene type B (PP) by scanning electron microscopy (SEM). The biofilms were developed at 30 °C for 240 h. (**A**) Lm-303 TSB; (**B**) Lm-320 in 1/10 diluted TSB; (**C**) Lm-356 in TSBA.

Furthermore, *L. monocytogenes* biofilms on PP were observed as cells irreversibly attached and microcolonies of metabolically active cells examined by an epifluorescence microscope (Figure 1). Mono-species *L. monocytogenes* showed that cells were linked and embedded in dense EPS (Figure 2).

### 3.3. Quantification and Components of the Matrix Biofilm Formation

**Figure 3:**
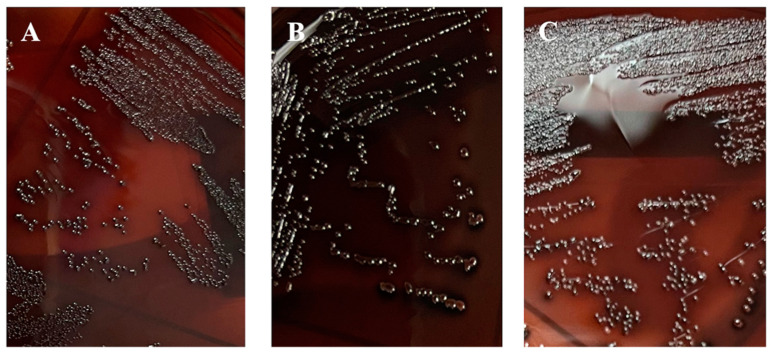
Black colonies of the slime-producing *L. monocytogenes*: (**A**) Lm-352; (**B**) Lm-303; (**C**) Lm-356.

DNase I and proteinase K were used to determine the components that make up the matrix of the *L. monocytogenes* biofilm; we found eDNA (9.44–47.69%) and protein (16.90–46.82%) (*p* < 0.05). In turn, there was a difference between each of the *L. monocytogenes* strains that made up the groups (*p* < 0.05) (Table 5). It was determined that 100% of *L. monocytogenes* strains were biofilm producers on CRA (Figure 3).

## 4. Discussion

In recent years, the consumption of fruit and vegetables has increased; consequently, there have been *L. monocytogenes* outbreaks associated with foods such as enoki mushrooms, cantaloupe, frozen vegetables, and packaged salads [7]. In addition, *L. monocytogenes* has the ability to develop biofilms in a food environment [21]. Sixty percent of outbreaks are caused by biofilm-associated infections by pathogens and antimicrobial resistance, which have a significant impact on the food industry [32,33].

In our study, the analysis of serogroups by PCR revealed that 66.6% of the *L. monocytogenes* isolates from Hass avocado were identified as serogroup III (1/2b, 3b, and 7), 27.7% as IV (4b, 4d, and 4e), and 5.5% as serogroup I (1/2a and 3a). Other investigators have demonstrated a higher prevalence of some serotypes (1/2b, 3b, 1/2a, 1/2c, and 4b) in food or processing environments [20,25,34,35]. According to the CDC (2011), serotypes 1/2a and 1/2b were associated with 147 cantaloupe-associated listeriosis cases in 28 U.S. states in 2011 [36]. This could be relevant for food safety and public health, as it shows the diversity of serogroups of *L. monocytogenes* in Hass avocados. It is essential to confirm the serotype with the incorporation of techniques used for subtyping *L. monocytogenes* such as random amplification of polymorphic DNA-polymerase chain reaction (RAPD-PCR), repetitive extragenic palindromes-PCR (REP-PCR), and pulsed field gel electrophoresis (PFGE), which allow for the sources and mechanisms of contamination during food processing to be determined and fresh food produce to be commercialized, at the same time identifying serotypes related to disease outbreaks. Likewise, Roche et al. [37] argued that the virulence of *L. monocytogenes* is related to its serotype and lineage (I–IV).

Additionally, 38.8% of *L. monocytogenes* isolates had virulence-associated genes (LIPI-1) (Table 2). Montero et al. [25] and Vilchis-Rangel et al. [38] reported similar percentages (20–40%) for LIPI-1 in *L. monocytogenes* isolates from orange juice, vegetables, and frozen vegetables. Moreover, several studies have reported LIPI-1 in 100% of *L. monocytogenes* isolates from duck, beef, pork, chicken, vegetables, fried rice, fish, goat meat, pasteurized milk, and yogurt [13,39,40]. The wide range of virulence-associated *L. monocytogenes* genes found in this study could be associated with food type, sources, mechanisms of contamination during food processing and marketing, epidemiological factors, countries, geographical differences, the genetic diversity of strains, and methodologies for the serotyping of *L. monocytogenes* in food. Overall, 55.5% of *L. monocytogenes* isolates carried genes corresponding to LIPI-1. However, 5.5% of *L. monocytogenes* did not carry LIPI-1 in this study (Table 2). These results agree with those of Montero et al. [25] and Vilchis-Rangel et al. [38], who reported that virulence-associated genes (LIPI-1) were not detected in *L. monocytogenes* isolates collected from frozen vegetables, orange juice, and fresh vegetables. Montero et al. [25] argued that the absence of one of these genes did not imply that a strain was not virulent. *L. monocytogenes* strains are known to differ in virulence [20]. For example, multiple distinct genetic mechanisms (mutations in the *inlA* and *prfA genes*) could be responsible for natural virulence attenuation in *L. monocytogenes* [41].

Biofilm formation-associated genes including *prfA, actA*, *inlA*, and *plcA* that play a significant role in the survival and persistence of *L. monocytogenes* were analyzed in this study (*n* = 12, 66.6%; *n* = 16, 88.8%; *n* = 6, 33.3%; *n* = 16, 88.8%). Likewise, Kumar et al. [42] showed that the presence of *hly*, *prfA*, and LIPI-1 genes is required to develop biofilm by *L. monocytogenes*. However, non-detection of *prfA* is associated with an L. monocytogenes prfA* mutant, which has decreased virulence [43].

In this study, *L. monocytogenes* isolates from Hass avocados had a high capacity for biofilm formation in supplement medium (TSBA, 1/10 diluted TSBA) and TSB in comparison with 1/10 diluted TSB (*p* < 0.05) at 48–240 h. Similarly, other studies have reported a high capacity for biofilm formation on polystyrene by *L. monocytogenes* isolated from cheese-processing plants, cheeses, and milk samples [26,27,44]. In contrast to our results, Kadam et al. [45] demonstrated that *L. monocytogenes* biofilm production was higher in a minimal medium compared to a nutrient-rich medium. This could be due to the organic matter or food residues that favored preconditioning and irreversible adhesion for the formation of biofilm. In fact, several studies have reported that the adherence and formation of biofilm by *L. monocytogenes* on surfaces is affected by factors such as temperature, pH, medium, incubation time, strain, serotype, and certain fatty acids (iso-C_14:0_, anteiso-C_15:0_, and iso-C_16:0_) [46,47].

Accordingly, the development of the biofilm of *L. monocytogenes* was favored at 240 h (*p* < 0.05) in this study. The difference was associated with several factors. First, consider that in 48 h at 30 °C, it is possible that *L. monocytogenes* isolates are developing an irreversible adhesion phase or the formation of microcolonies is occurring, compared to at 240 h, where the cell density has increased and bacteria would be in the stage of maturation or dispersal. Researchers argue that the biofilm formation capacity of *L. monocytogenes* increases after 72 h and the biofilm maturation stage occurs at 240 h [48,49]. Several studies have argued for a possible correlation between the serotype or phylogenetic division and biofilm-forming ability [22,50,51]. In addition, a CV staining assay is used for biofilm biomass quantification, but does not reveal the number of viable cells within the biofilm matrix [52,53].

In this study, *L. monocytogenes* in mono-species biofilms (Lm-303, Lm-320, Lm-352, and Lm-356) had a higher cellular density in TSBA (7.78 ± 0.03–8.82 ± 0.03 log_10_ CFU cm^−2^; *p* < 0.05) in comparison with TSB, 1/10 diluted TSB, and 1/10 diluted TSBA at 240 h onto polypropylene type B (Table 4). In particular, Lm-133 had a lower cellular density in TSB, 1/10 diluted TSB, and TSBA in comparison with Lm-320 (*p* < 0.05). This may be associated with the biofilm formation capacity of this particular serotype, even though it is serogroup III (1/2b, 3b, and 7), and with intrinsic factors like the nutrient level in the culture medium. In addition, this agrees with previous studies demonstrating the biofilm-forming ability of *L. monocytogenes* on materials encountered in the food-processing industry, such as stainless steel, aluminum, polycarbonate, polypropylene, polyurethane, polyvinylchloride, silicone rubber, natural white rubber, PETG, PTFE, Lexan, Nitryl rubber, and glass [17,54].

However, Pan et al. [55] and Nilsson et al. [56] revealed that the serotype or strain origin can have an effect on the biofilm-forming behavior of *L. monocytogenes*. Hence, a biofilm is a complex system where the different stages of biofilm formation alternate, depending on many factors such the composition of the medium or different levels of nutrients, which can influence the cell–cell communication; this drives the physiological and metabolic processes within the biofilm and affects biofilm development [50,57]. In addition, the biofilm matrix contains one or more extracellular polymeric substances (EPS) such as polysaccharides, proteins, extracellular DNA (eDNA), lipids, polyglutamate, teichoic acids, humic substances, etc. [33,58,59].

Our epifluorescence microscopy results revealed the metabolically active cells in the microcolonies formed (Figure 1), and SEM was used to assess the biofilm architecture (EPS and embedded bacterial cells) (Figure 2) onto polypropylene type B. In particular, treatment with DNase I and proteinase K revealed the presence of proteins (16.90–46.82%) and eDNA (9.44–47.69%). In addition, on CRA, 100% of the *L. monocytogenes* isolates were biofilm producers, because the extracellular polysaccharides combine with the Congo red dye, demonstrating exopolysaccharides of *L. monocytogenes* (Figure 3).

These results agree with those of Jiao et al. [60] and Muthukrishnan et al. [61], who showed that extracellular proteins are a major EPS components of the biofilm dry mass. Likewise, Kadam et al. [45] showed the presence of eDNA after DNase I was added to the microtiter plates during biofilm formation by *L. monocytogenes* (strains 18 and 55). Recently, studies have reported the structural components of *L. monocytogenes* within the EPS, such as eDNA, proteins (InlA, BapL, PlcA, FlaA, PBP, and ActA), polysaccharides (poly-β-(1-4)-N-acetylmannosamine (poly-NAM), and teichoic acids (WTA and LTA)); moreover, their roles in bacterial adhesion and aggregation, and as a structural component within the biofilm that provides stability to the entire structure and horizontal gene transfer, have been revealed [18,62,63,64,65].

Additionally, EPS strengthen the survival of microorganisms embedded on a substratum; moreover, they decrease the effectiveness of disinfectants, having a similar effect as organic matter or food residues present on the surface, resulting in less disinfectant coming into contact with the microorganism and reducing the effectiveness of the disinfectant or antimicrobial agent [66,67,68,69,70]. Our results emphasize the importance of incorporating other types of materials into the food industry so that they do not allow for the adherence and formation of biofilms in fresh produce. However, the design of strategies for the prevention and/or removal of biofilms of *L. monocytogenes* will also be necessary. In addition, techniques such as confocal laser scanning microscopy (CLSM) to reveal the structural components that make up the biofilm matrix and serotyping of *L. monocytogenes* can be used to determine the sources and mechanisms of contamination during the processing and marketing of fresh food products.

## 5. Conclusions

*L. monocytogenes* has the ability to develop a biofilm that harbors virulence-associated genes (LIPI-1) of *L. monocytogenes* isolates from Hass avocados. This could be a relevant food safety and public health consideration. However, serotyping is required to determine the prevalence, severity, and association of serotypes of *L. monocytogenes* with the ability to develop biofilm. Our study showed that the development of biofilms of *L. monocytogenes* is affected by 1/10 diluted TSB at 48–240 h using a CV staining assay. However, the development of biofilms of *L. monocytogenes* had a higher cellular density in TSBA onto PP. Additionally, future research should consider the detection of genes involved in other islands of pathogenicity (LIPI-2, LIPI-3, and LIPI-4) of *L. monocytogenes* isolates from Hass avocados.

## Figures and Tables

**Table 1:** Primers used in this study.

Primers	Length (bp)	Sequence 5′—3′		Tm (°C)
*plcB*	723	Forward	5′-CAG CTC CGC ATG ATA TTG AC-3′	58
		Reverse	5′-CTG CCA AAG TTT GCT GTG AA-3′	
*Inl 1A*	629	Forward	5′-GGC TGG GCA TAA CCA AAT TA-3′	60
		Reverse	5′-CTT TTG GTG CCG TAG GT-3′	
*Inl 1B*	293	Forward	5′-CCT AAA CCT CCG ACC AAA CA-3′	60
		Reverse	5′-CCA TTT CGC GCT TCT CTA TC-3′	
*prfA*	330	Forward	5′-ACC AAT GGG ATC CAC AAG AA-3′	58
		Reverse	5′-GCT TCC CGT TAA TCG AAA AAT-3′	
*icA*	840	Forward	5′-TCC CAT TAG GTG GAA AAG CA-3′	57
*plcA*		Reverse	5′-CGG GGA AGT CCA TGA TTA GA-3′	
*Hly*	1100	Forward	5′-GTC TAC CAA TTG CGC AAC AA-3′	57
		Reverse	5′-TGG TGT TTC CCG GTT AAA AG-3′	
*Mpl*	450	Forward	5′-AAA GGT GGA GAA ATT GAT TCG-3′	62
		Reverse	5′-AGT GAT CGT ATT GTA GGC TGC TT-3′	
*actA*	571	Forward	5′-AAA CAG AAG AGC CAA GC-3′	58
		Reverse	5′-TTC ACT TCG GGA TTT TCG TC-3′	

**Table 2:** Serogroups and genetic determinants of virulence of *Listeria monocytogenes*.

PCR-Serogroups	Strain ID	Genetic Determinants of Virulence No. of Isolates (%)							
		*prfA*	*plcA*	*hly*	*mpl*	*actA*	*plcB*	*inlB*	*inlA*
I	Lm-334	+	−	+	+	+	−	−	+
III	Lm-132	−	−	−	−	−	−	+	−
	Lm-237	−	+	−	+	+	−	+	−
	Lm-249	+	+	+	+	+	−	+	−
	Lm-252	−	+	+	+	+	−	+	+
	Lm-253	+	+	+	+	+	−	+	−
	Lm-304 *	+	+	+	+	+	+	+	−
	Lm-333 *	+	+	+	+	+	+	+	−
	Lm-335	+	+	−	+	+	+	−	−
	Lm-336 *	+	+	+	+	+	+	+	+
	Lm-352 *	+	+	+	+	+	+	−	+
	Lm-356 *	+	+	+	+	+	+	+	−
	Lm-357	+	+	+	−	+	+	+	−
IV	Lm-142	−	+	−	−	−	−	−	−
	Lm-251	−	+	+	+	+	-	+	+
	Lm-303 *	+	+	+	+	+	+	+	−
	Lm-320 *	+	+	+	+	+	+	+	−
	Lm-332	−	+	+	+	+	+	+	+
Total	18 (100%)	12 (66.6%)	16 (88.88%)	14 (77.7%)	15 (83.3%)	16 (88.8%)	10 (55.5%)	14 (77.7%)	6 (33.3%)

* Positive isolates of all genes present in the *Listeria* pathogenicity island 1 (LIPI-1); Lm, *Listeria monocytogenes*. +, presence; – absence.

**Table 3:** Biofilm formation by *Listeria monocytogenes* in different medium cultures and temperatures.

Time	Medium Culture	No. of Strains (%) Polystyrene Microtiter Plates Biofilm Formation			
		Strong Biofilm	Moderate Biofilm	Weak Biofilm	Non-Biofilm
		Producers	Producers	Producers	Producers
48 h	TSBA^a^	4 (22.2)	0	7 (38.8)	7 (38.8)
		OD_570_ mean = 0.346 ± 0.007		OD_570_ mean = 0.091 ± 0.007	OD_570_ mean = 0.075 ± 0.000
	1/10 diluted TSBA^b^	4 (22.2)	0	14 (77.7)	0
		OD_570_ mean = 0.289 ± 0.002		OD_570_ mean = 0.076 ± 0.004	
	TSB^c^	0	1 (5.5)	17 (94.4)	0
			OD_570_ mean = 0.210 ± 0.014	OD_570_ mean = 0.093 ± 0.018	
	1/10 diluted TSB^d^	0	4 (22.2)	14 (77.7)	0
			OD_570_ mean = 0.125 ± 0.006	OD_570_ mean = 0.072 ± 0.002	
240 h	TSBA^a^	6 (33.3)	12 (66.6)	0	0
		OD_570_ mean = 0.402 ± 0.078	OD_570_ mean = 0.218 ± 0.082		
	1/10 diluted TSBA^b^	11 (61.1)	7 (38.8)	0	0
		OD_570_ mean = 0.317 ± 0.013	OD_570_ mean = 0.183 ± 0.018		
	TSB^c^	1 (5.5)	17 (94.4)	0	0
		OD_570_ mean = 0.302 ± 0.007	OD_570_ mean = 0.226 ± 0.046		
	1/10 diluted TSB^d^	3 (16.6)	15 (83.3)	0	0
		OD_570_ mean = 0.275 ± 0.004	OD_570_ mean = 0.183 ± 0.011		

TSB: tryptic soy broth; 1/10 diluted TSB; TSBA (TSB with 1% Hass avocado residues); 1/10 diluted TSBA (1/10 TSB + 1% Hass avocado residues); OD_570_ values ± SDs obtained by polystyrene biofilm formation assays in the corresponding culture medium. The cutoff OD (ODc): TSBA^a^ OD570 ≤ ODc_570_ 0.078 = non-biofilm producers; 0.078 < OD_570_ ≤ 0.156 = weak biofilm; 0.156 < OD_570_ ≤ 0.312 = moderate biofilm producers; and 0.312 < OD_570_ = strong biofilm; 1/10 diluted TSBA^b^ OD_570_ ≤ ODc_570_ 0.059 = non-biofilm producers; 0.059 < OD_570_ ≤ 0.118 = weak biofilm; 0.118 < OD_570_ ≤ 0.236 = moderate biofilm producers; and 0.236 < OD_570_ = strong biofilm; TSB^c^ OD_570_ ≤ ODc_570_ 0.07 = non-biofilm producers; 0.07 < OD_570_ ≤ 0.14 = weak biofilm; 0.14 < OD_570_ ≤ 0.28 = moderate biofilm producers; and 0.28 < OD_570_ = strong biofilm; 1/10 diluted TSB^d^ OD_570_ ≤ ODc_570_ 0.057 = non-biofilm producers; 0.057 < OD_570_ ≤ 0.114 = weak biofilm; 0.114 < OD_570_ ≤ 0.228 = moderate biofilm producers; and 0.228 < OD_570_ = strong biofilm.

**Table 4:** Cellular density of *L. monocytogenes* in mono-species biofilms.

Mono-Species Biofilms	Culture Media	Log_10_ CFU/cm^2^ ± SD ^A^
		Polypropylene Type ^B^
Lm-303	TSB	7.54 ± 0.08 ^g^
	1/10 diluted TSB	7.52 ± 0.06 ^g^
	TSBA	8.36 ± 0.01 ^b^
	1/10 diluted TSBA	7.54 ± 0.05 ^fg^
Lm-320	TSB	7.60 ± 0.08 ^efg^
	1/10 diluted TSB	7.59 ± 0.14 ^efg^
	TSBA	8.82 ± 0.03 ^a^
	1/10 diluted TSBA	7.49 ± 0.07 ^g^
Lm-352	TSB	7.61 ± 0.12 ^defg^
	1/10 diluted TSB	7.56 ± 0.09 ^efg^
	TSBA	7.78 ± 0.03 ^cdefg^
	1/10 diluted TSBA	7.66 ± 0.06 ^defg^
Lm-356	TSB	7.67 ± 0.10 ^defg^
	1/10 diluted TSB	7.61 ± 0.12 ^defg^
	TSBA	7.84 ± 0.30 ^cdef^
	1/10 diluted TSBA	7.61 ± 0.11 ^efg^
*Listeria monocytogenes* ATCC 19111	TSB	7.66 ± 0.30 ^defg^
	1/10 diluted TSB	7.92 ± 0.59 ^cd^
	TSBA	8.01 ± 0.09 ^c^
	1/10 diluted TSBA	7.85 ± 0.18 ^cde^

^A^ Values are the means of three tests ± standard deviation. ^B^ Values in the same column followed different lowercase letters are significantly different (*p* < 0.05). TSB: tryptic soy broth; TSBA: TSB with 1% avocado residues; 1/10 diluted TSBA: 1/10 diluted TSB with 1% avocado residues.

**Table 5:** Biomass reduction of mono-species biofilms of *Listeria monocytogenes* isolates of Hass avocados.

Serogroups	Mono-Species Biofilms	Biomass Reduction (%)	
		Proteinase K	DNase I
I	Lm-334	36.80 ± 0.10 ^e^	47.69 ± 0.04 ^a^
III	Lm-132	38.98 ± 0.03 ^d^	7.86 ± 0.06 ^p^
	Lm-237	32.67 ± 0.07 ^h^	28.96 ± 0.01 ^e^
	Lm-249	19.80 ± 0.07 ^m^	6.67 ± 0.00 ^r^
	Lm-252	18.38 ± 0.04 ^o^	11.91 ± 0.00 ^m^
	Lm-253	12.48 ± 0.02 ^q^	13.35 ± 0.17 ^k^
	Lm-304	16.90 ± 0.11 ^p^	14.59 ± 0.02 ^j^
	Lm-333	34.79 ± 0.03 ^g^	9.75 ± 0.04 ^n^
	Lm-335	25.33 ± 0.01 ^j^	44.54 ± 0.21 ^c^
	Lm-336	26.18 ± 0.02 ^i^	44.87 ± 0.06 ^b^
	Lm-352	40.82 ± 0.07 ^b^	18.85 ± 0.03 ^g^
	Lm-356	35.36 ± 0.01 ^f^	9.46 ± 0.00 ^o^
	Lm-357	46.82 ± 0.06 ^a^	16.95 ± 0.03 ^h^
IV	Lm-142	39.55 ± 0.06 ^c^	33.08 ± 0.00 ^d^
	Lm-251	19.23 ± 0.07 ^n^	7.56 ± 0.03 ^q^
	Lm-303	20.92 ± 0.03 ^l^	23.72 ± 0.00 ^f^
	Lm-320	23.60 ± 0.11 ^k^	9.44 ± 0.0287 ^o^
	Lm-332	16.92 ± 0.01 ^p^	12.83 ± 0.06 ^l^
*Listeria monocytogenes* ATCC 19111		46.80 ± 0.01 ^a^	15.87 ± 0.01 ^i^

Values are expressed as the means of three tests ± standard deviation. Values in the same column followed by different lowercase letters are significantly different (*p* < 0.05).

## Data Availability

The data used to support the findings of this study are available from the corresponding author upon request.
